# Supplementary material for: Cryo-EM structure of TMEM63C suggests it functions as a monomer
Source: Nat Commun. 2023 Nov 9;14:7265. doi: 10.1038/s41467-023-42956-2 (PMC10636204; doi:10.1038/s41467-023-42956-2)
Supplement: Supplementary file 1 — Supplementary Information [file 41467_2023_42956_MOESM1_ESM.pdf]

Supplementary Information for

Cryo-EM structure of TMEM63C suggests it functions as a monomer

Yuqi Qin[1,8], Daqi Yu[1,8], Dan Wu[2,8], Jiangqing Dong[1,8], William Thomas Li[1], Chang Ye[2], Kai Chit Cheung[1], Yingyi Zhang[3], Yun Xu[2], YongQiang Wang[4,*], Yun Stone Shi[2,5,*], Shangyu Dang[1,6,7,*]

*Correspondence: yongqiang.wang@ucsf.edu (Y.W.); shiyun@gdiist.cn (Y.S.S); sdang@ust.hk (S.D.)

This file includes:

- Supplementary Figures 1 to 9
- Supplementary Table 1

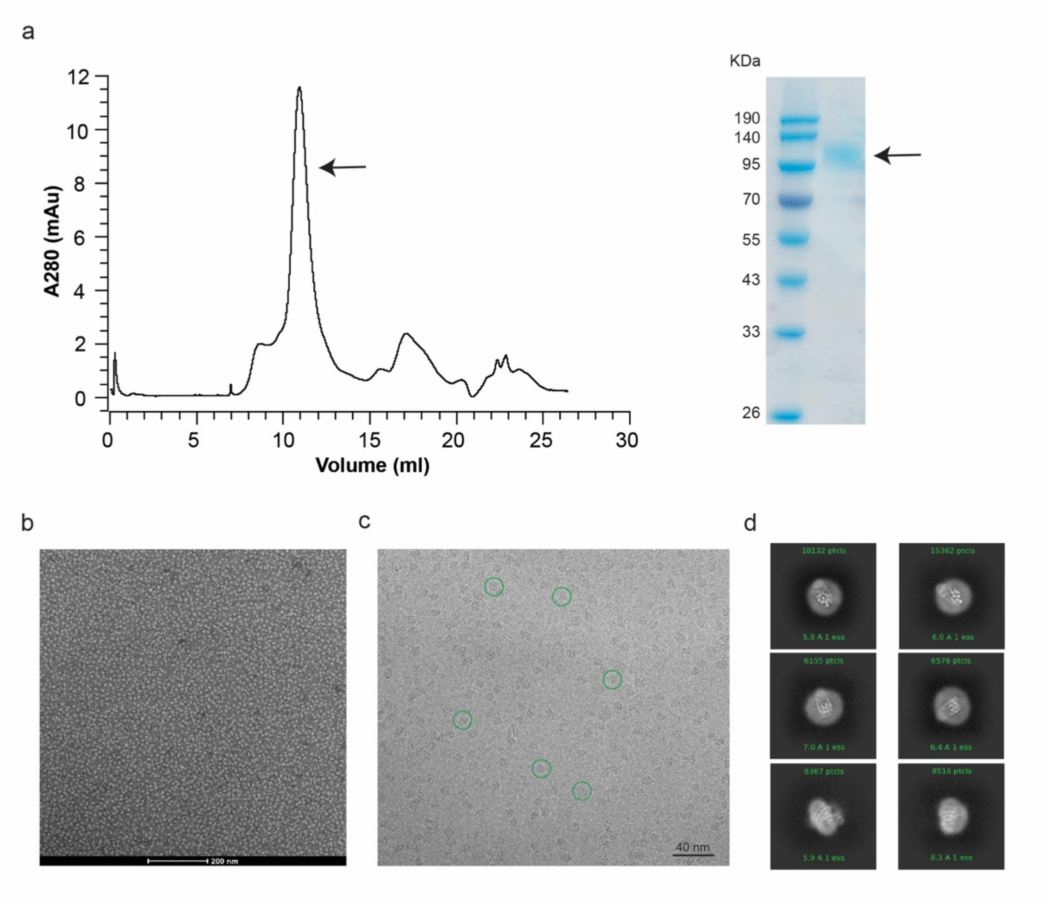

Supplementary Figure 1 Sample preparation of TMEM63C. (**a**) Size-exclusion chromatography of TMEM63C in detergent LMNG (left). The peak fraction is verified by Coomassie blue staining SDS-PAGE (right). (**b**) A representative negative stain micrograph of TMEM63C. (**c**). A representative cryo-EM micrograph of TMEM63C. Green circles indicate individual particles of TMEM63C. (**d**) The 2D class average of TMEM63C final reconstituted particles.

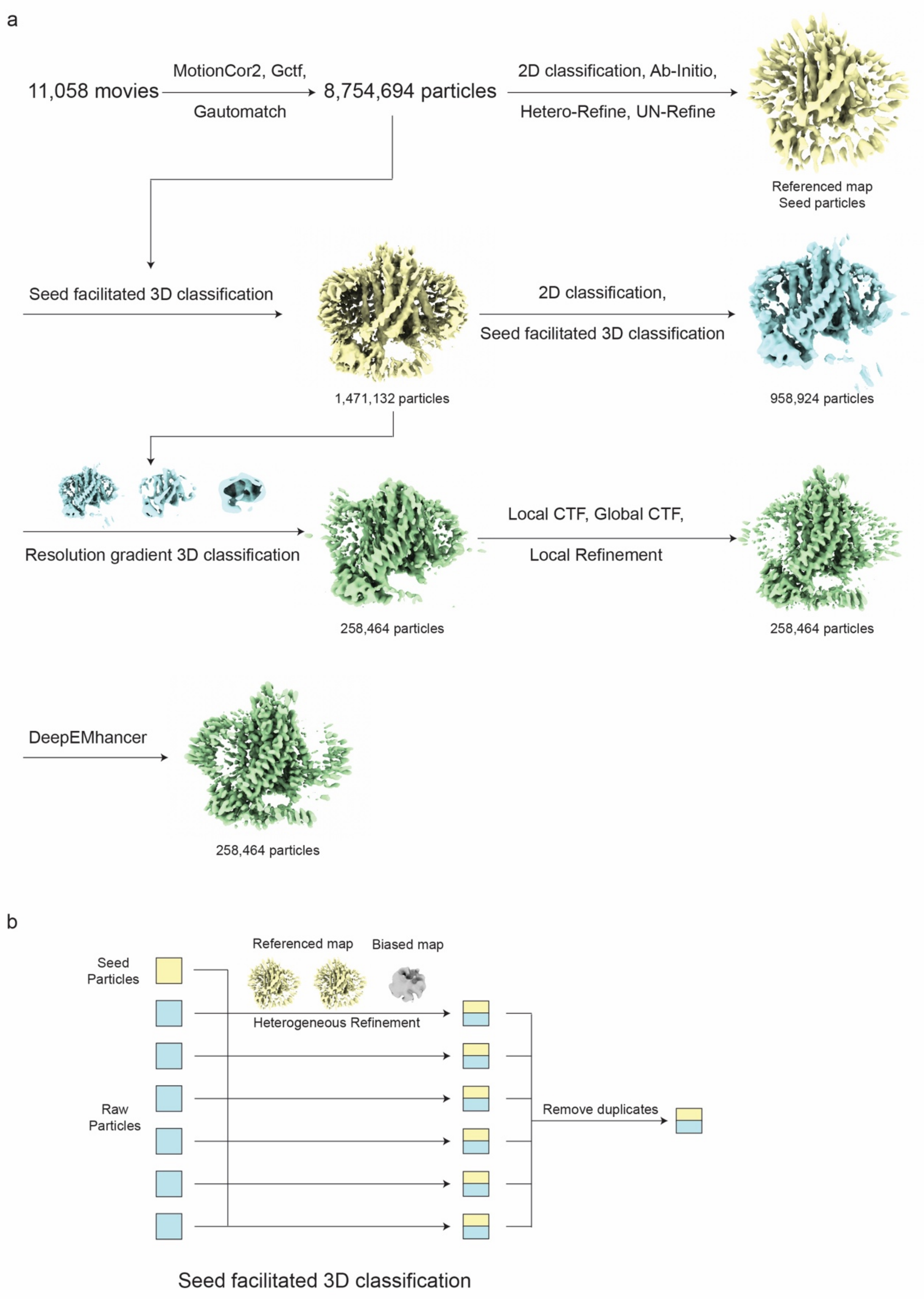

Supplementary Figure 2 Cryo-EM data processing. (**a**) Workflow of the TMEM63C data processing. (**b**) Details of seed facilitated 3D classification.

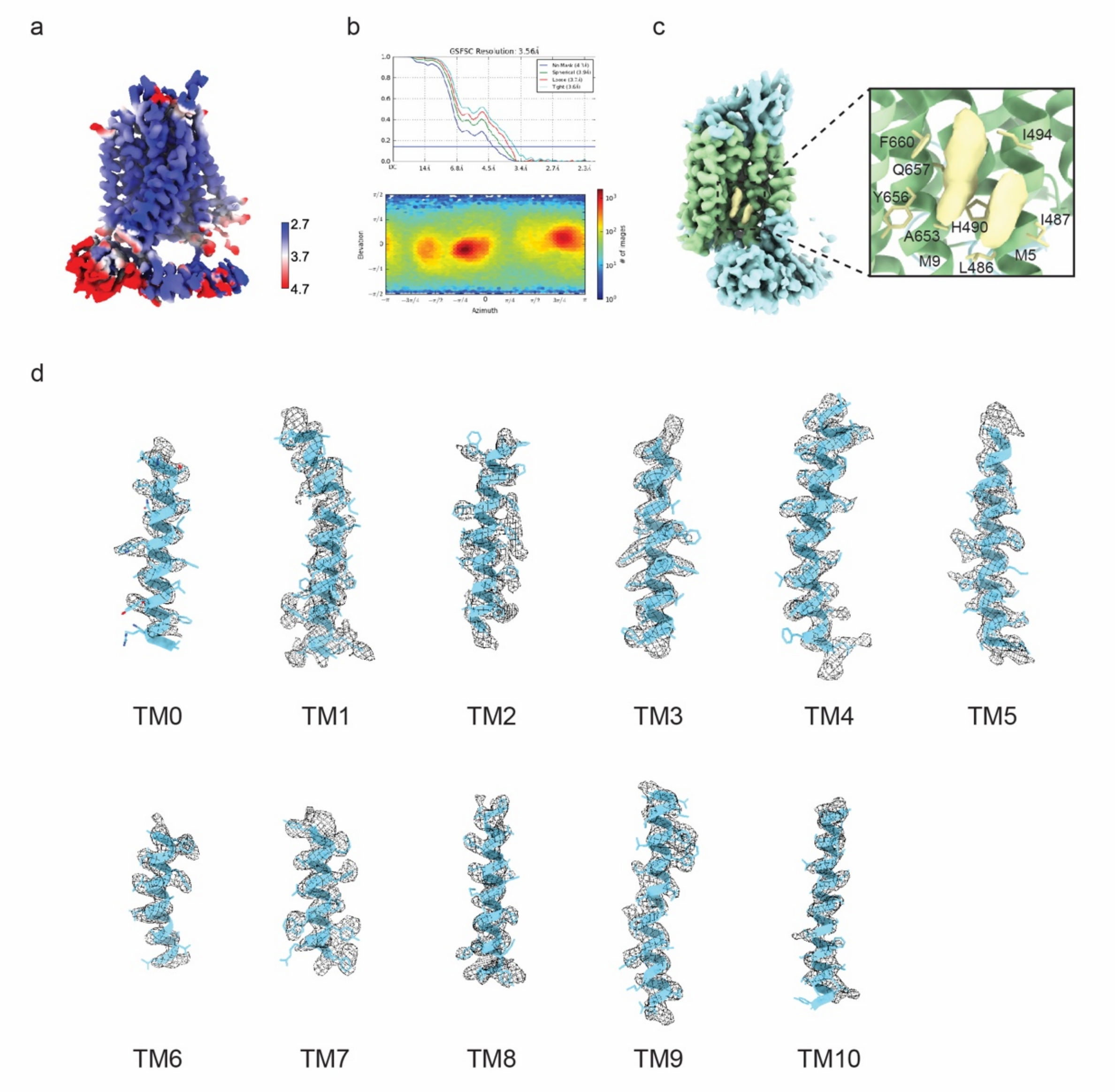

Supplementary Figure 3 Cryo-EM analysis of TMEM63C. (**a**) Local resolution estimation of TMEM63C density map. (**b**) FSC curves (top) and angular distribution (bottom) of the 3D reconstruction of TMEM63C at 3.56 Å resolution. (**c**) Side view of TMEM63C. The lipid-like density is colored in yellow. The lipid surrounding residues in TM5 and TM9 are shown as yellow. (**d**) Cryo-EM density segments (black mesh) and atomic models (blue) of 11 transmembrane α-helices.

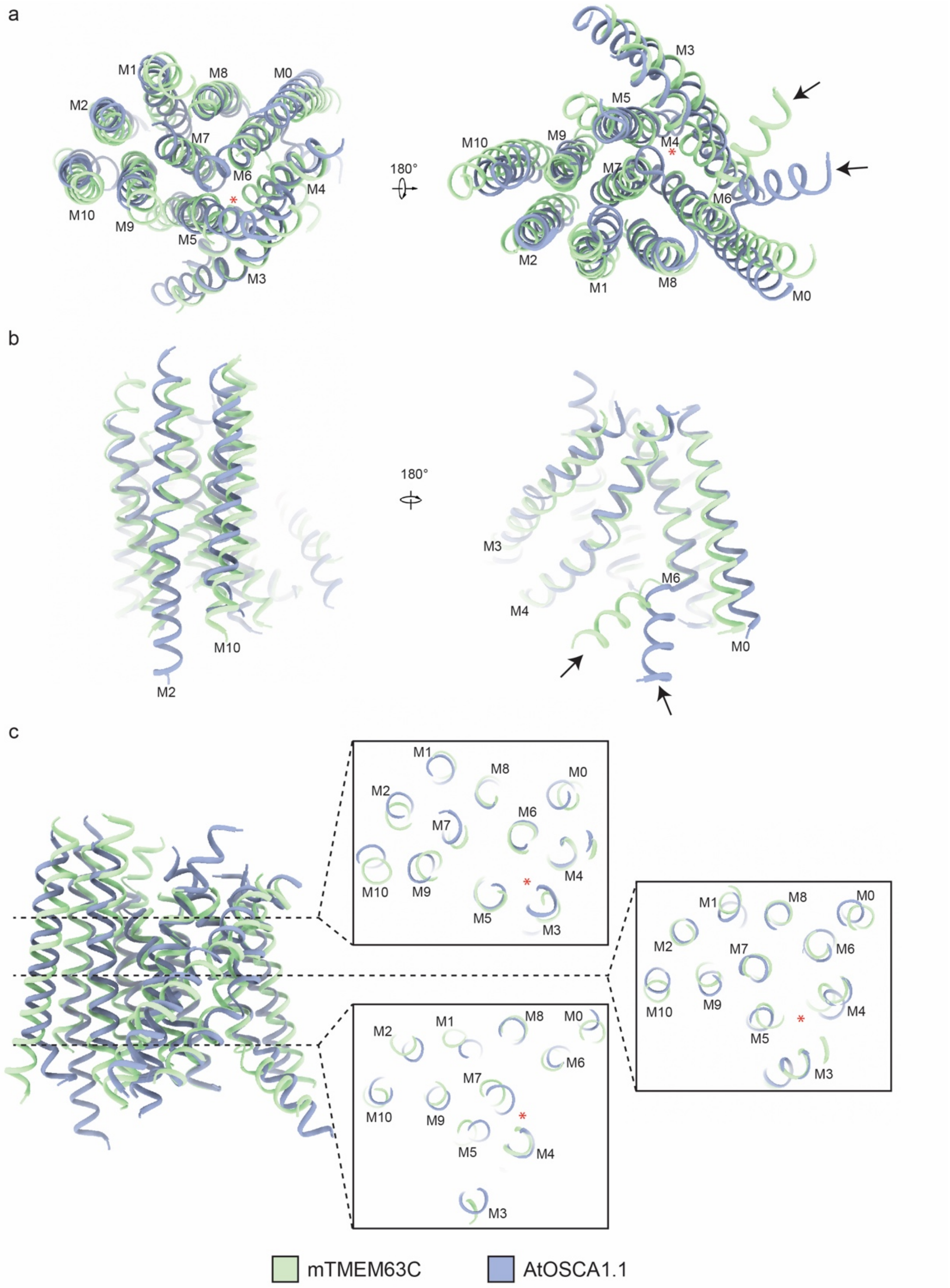

Supplementary Figure 4 Structure comparison of mTMEM63C and AtOSCA1.1 (PDB:6JPF). (**a**) Top view (left) and bottom view (right) of mTMEM63C (green) and AtOSCA1.1 (dusty blue). The eleven transmembrane α-helices are labeled with M0 to M10. (**b**) Side views of mTMEM63C and AtOSCA1.1. The M2, M10 and α-helices linked to M6 (black arrows) show conformation changes between mTMEM63C and AtOSCA1.1. (**c**) Cross sections of mTMEM63C and AtOSCA1.1. The red asterisk indicates the pore region.

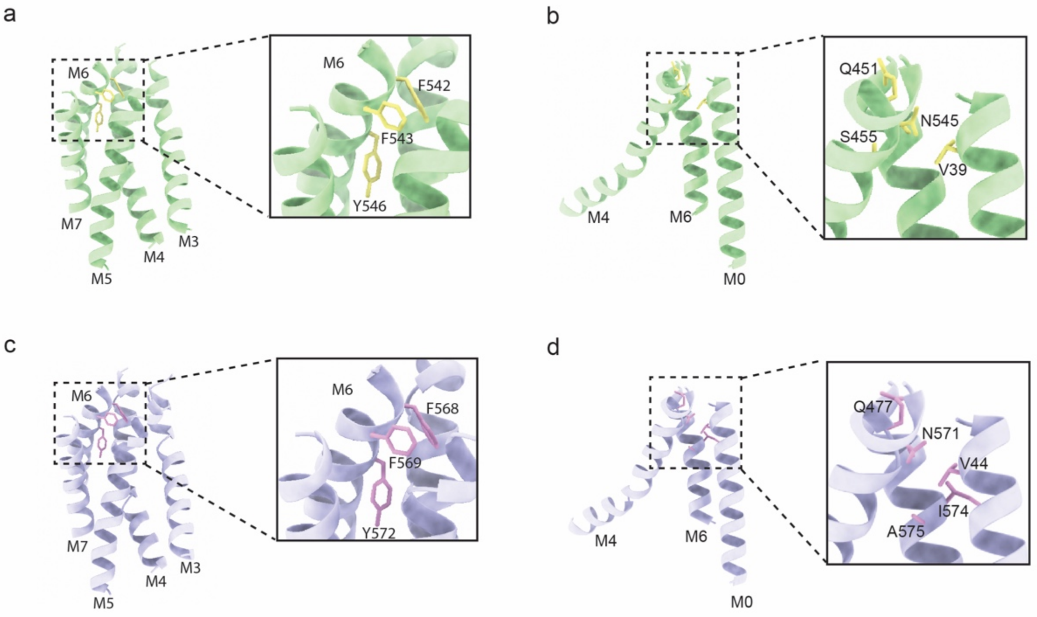

Supplementary Figure 5 Key residues that are important for gating in TMEM63C and TMEM63B. (**a**) TMEM63C pore forming α-helices TM3 to TM7 (green). The conserved bulky residues F542, F543 and Y546 (yellow) of TMEM63C in the pore. (**b**) TMEM63C V39 in TM0, Q451, S455 in TM4 and N545 in TM6. (**c**) TMEM63B pore forming α-helices M3 to M7 (purple). The conserved bulky residues F568, F569 and Y572 (pink) of TMEM63B in the pore. (**d**) TMEM63B V44 in TM0, Q477 in TM4, N571, I574 and A575 in TM6. All the residues are near the extracellular side.

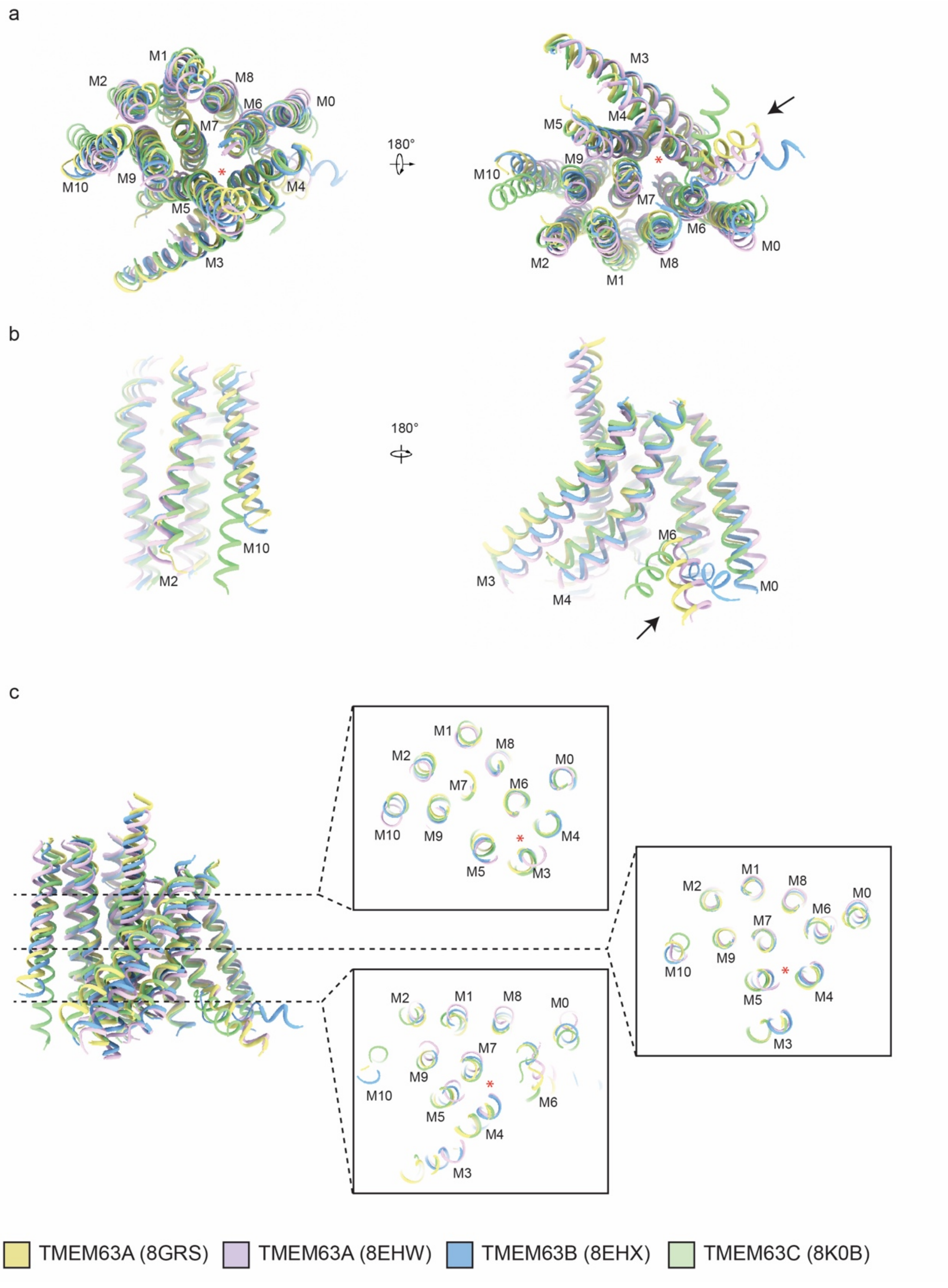

Supplementary Figure 6 Structural comparison of TMEM63s. (**a**) Top view (left) and bottom view (right) of structural comparison including TMEM63A (yellow, PDB: 8GRS), TMEM63A (pink, PDB: 8EHW), TMEM63B (blue, PDB: 8EHX), and TMEM63C (green, PDB: 8K0B). The eleven transmembrane α-helices are labeled with M0 to M10. (**b**) Side views of TMEM63. The M10, M3, M4 and M6b (black arrows) show conformation changes. (c) Cross sections of structural comparison of TMEM63 proteins. The red asterisk indicates the pore region.

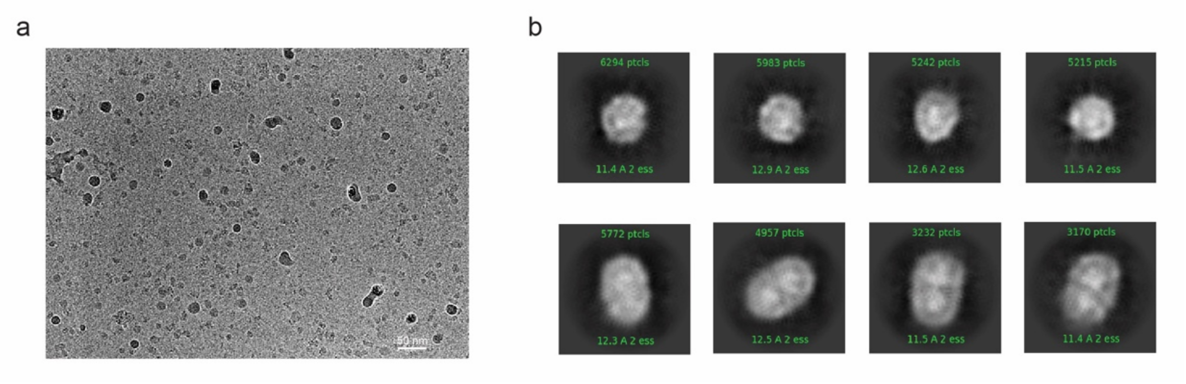

Supplementary Figure 7 Cryo-EM analysis of TMEM63B. (**a**) A representative cryo-EM micrograph of TMEM63B. The scale bar is 50nm. (**b**) The 2D class averages of TMEM63B indicate that both monomer (top) and dimer (bottom) exist.

Similarity / Identity	mTMEM63C	mTMEM63B	mTMEM63A	HsTMEM63C	HsTMEM63B	HsTMEM63A	DmTMEM63	OsOSCA1.2	AtOSCA1.1	AtOSCA1.2	AtOSCA3.1
mTMEM63C	100	63	60	91	62	63	51	40	39	39	41
mTMEM63B	44	100	75	62	98	75	53	44	41	41	40
mTMEM63A	41	58	100	60	75	95	52	42	40	40	40
HsTMEM63C	84	42	40	100	62	64	51	42	40	41	39
HsTMEM63B	43	98	58	43	100	75	54	44	41	42	41
HsTMEM63A	44	59	90	44	59	100	52	42	39	39	39
DmTMEM63	31	33	32	31	33	31	100	41	41	42	39
OsOSCA1.2	24	25	24	24	25	24	21	100	80	81	53
AtOSCA1.1	22	21	21	22	22	22	21	69	100	92	52
AtOSCA1.2	19	21	21	22	21	22	20	69	85	100	51
AtOSCA3.1	23	24	25	21	24	23	22	33	30	29	100

Supplementary Figure 8 Summary of sequence identity (green) and similarities (blue) of proteins in OSCA/TMEM63 family. mTMEM63C (UniProtKB: Q8CBX0), mTMEM63B (UniProtKB: Q3TWI9), mTMEM63A (UniProtKB: Q91YT8), HsTMEM63C (UniProtKB: Q9P1W3), HsTMEM63B (UniProtKB: Q5T3F8), HsTMEM63A (UniProtKB: O94886), DmTMEM63 (UniProtKB: Q6NP91), OsOSCA1.2 (UniProtKB: Q5TKG1), AtOSCA1.1 (UniProtKB: Q9XEA1), AtOSCA1.2 (UniProtKB: Q5XEZ5) and AtOSCA3.1 (UniProtKB: Q9C8G5).

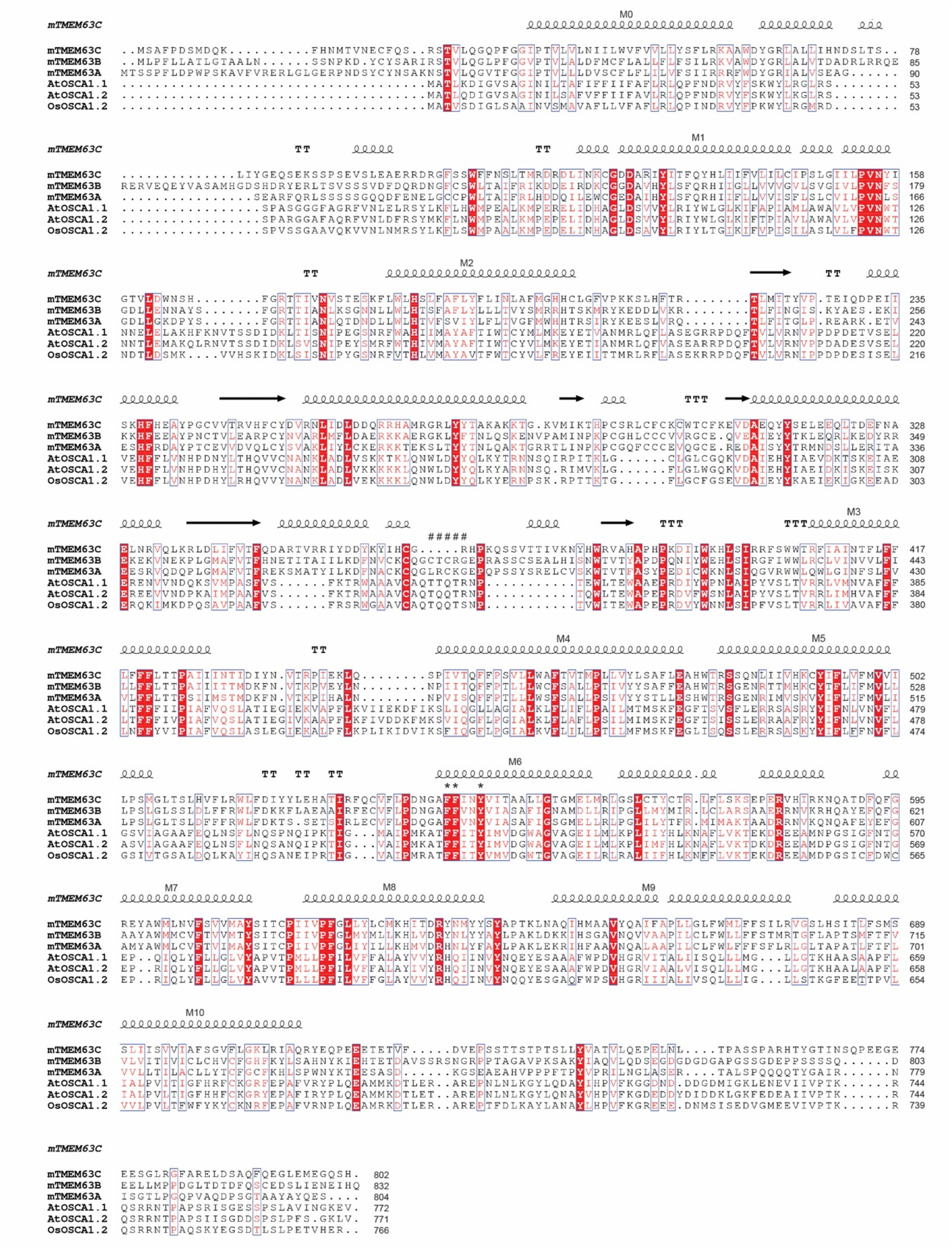

Supplementary Figure 9 Sequence alignment of mTMEM63C, mTMEM63B, mTMEM63A, AtOSCA1.1, AtOSCA1.2 and OsOSCA1.2. The conserved residues are colored in red. The secondary structure is shown above the sequences based on the mTMEM63C structure. α-helices are indicated as coil, β-sheets are indicated as arrows, and β-turns are indicated as T. The eleven transmembrane α-helices are

labeled with M0 to M10. The symbol “*” shows the three gating residues in the pore, and the symbol “#” shows the OSCA dimer interface residues.

Supplementary Table 1 Cryo-EM data collection, refinement and validation statistics

	**TMEM63C (EMDB-36759) (PDB 8K0B)**
**Data collection and processing**	
Magnification	81,000 x
Voltage (kV)	300
Electron exposure (e–/Å^2)	50
Defocus range (μm)	-2.5 ~ -1.0
Pixel size (Å)	1.06
Symmetry imposed	C1
Initial particle images (no.)	8,754,694
Final particle images (no.)	258,464
Map resolution (Å)	3.56
FSC threshold	0.143
Map resolution range (Å)	2.3 ~ 8.1
**Refinement**	
Initial model used (PDB code)	N/A
Model resolution (Å)	6.63
FSC threshold	0.5
Model resolution range (Å)	N/A
Map sharpening *B* factor (Å^2)	-113.5
Model composition	
Non-hydrogen atoms	5689
Protein residues	689
Ligands	N/A
B factors (Å^2)	
Protein	89.5
Ligand	N/A
R.m.s. deviations	
Bond lengths (Å)	0.004
Bond angles (°)	0.865
Validation	
MolProbity score	2.25
Clashscore	14.96
Poor rotamers (%)	1.28
Ramachandran plot	
Favored (%)	91.99
Allowed (%)	8.01
Disallowed (%)	0.00
