# Supplementary material for: Cryo-EM structure of TMEM63C suggests it functions as a monomer
Source: Nat Commun. 2023 Nov 9;14:7265. doi: 10.1038/s41467-023-42956-2 (PMC10636204; doi:10.1038/s41467-023-42956-2)
Supplement: Supplementary file 3 — Reporting Summary [file 41467_2023_42956_MOESM3_ESM.pdf]

nature portfolio

Corresponding author(s): YongQiang Wang, Yun Stone Shi, Shangyu Dang

Last updated by author(s): Oct 16, 2023

Reporting Summary

Nature Portfolio wishes to improve the reproducibility of the work that we publish. This form provides structure for consistency and transparency in reporting. For further information on Nature Portfolio policies, see our Editorial Policies and the Editorial Policy Checklist.

Statistics

For all statistical analyses, confirm that the following items are present in the figure legend, table legend, main text, or Methods section.

n/a	Confirmed	
☐	☒	The exact sample size (n) for each experimental group/condition, given as a discrete number and unit of measurement
☐	☒	A statement on whether measurements were taken from distinct samples or whether the same sample was measured repeatedly
☐	☒	The statistical test(s) used AND whether they are one- or two-sided<br>*Only common tests should be described solely by name; describe more complex techniques in the Methods section.*
☐	☒	A description of all covariates tested
☐	☒	A description of any assumptions or corrections, such as tests of normality and adjustment for multiple comparisons
☐	☒	A full description of the statistical parameters including central tendency (e.g. means) or other basic estimates (e.g. regression coefficient) AND variation (e.g. standard deviation) or associated estimates of uncertainty (e.g. confidence intervals)
☐	☒	For null hypothesis testing, the test statistic (e.g. F, t, r) with confidence intervals, effect sizes, degrees of freedom and P value noted<br>*Give P values as exact values whenever suitable.*
☒	☐	For Bayesian analysis, information on the choice of priors and Markov chain Monte Carlo settings
☒	☐	For hierarchical and complex designs, identification of the appropriate level for tests and full reporting of outcomes
☒	☐	Estimates of effect sizes (e.g. Cohen's d, Pearson's r), indicating how they were calculated

Our web collection on statistics for biologists contains articles on many of the points above.

Software and code

Policy information about availability of computer code

Data collection	EPU V2.10.0
Data analysis	MotionCor2 v1.4.0, GCTF, Gautomatch, RELION 3.1, cryoSPARC v2.15.0, UCSF Chimera v1.11.2, COOT, Phenix v1.20, GraphPad Prism 8.0

For manuscripts utilizing custom algorithms or software that are central to the research but not yet described in published literature, software must be made available to editors and reviewers. We strongly encourage code deposition in a community repository (e.g. GitHub). See the Nature Portfolio guidelines for submitting code & software for further information.

Data

Policy information about availability of data

All manuscripts must include a data availability statement. This statement should provide the following information, where applicable:

- Accession codes, unique identifiers, or web links for publicly available datasets
- A description of any restrictions on data availability
- For clinical datasets or third party data, please ensure that the statement adheres to our policy

Cryo-EM density map of TMEM63C has been deposited in the Electron Microscopy Data Bank under accession code EMD-36759 [https://www.ebi.ac.uk/emdb/EMD-36759]. Atomic coordinate has been deposited in the Protein Data Bank under accession code 8K0B [http://doi.org/10.2210/pdb8K0B/pdb]. All other data are available from the corresponding authors upon reasonable request. Source data are provided with this paper.

Research involving human participants, their data, or biological material

Policy information about studies with human participants or human data. See also policy information about sex, gender (identity/presentation), and sexual orientation and race, ethnicity and racism.

Reporting on sex and gender	Not applied to this study, which focuses on structural and functional analyses of TMEM63C.
Reporting on race, ethnicity, or other socially relevant groupings	Not applied to this study, which focuses on structural and functional analyses of TMEM63C.
Population characteristics	Not applied to this study, which focuses on structural and functional analyses of TMEM63C.
Recruitment	Not applied to this study, which focuses on structural and functional analyses of TMEM63C.
Ethics oversight	Not applied to this study, which focuses on structural and functional analyses of TMEM63C.

Note that full information on the approval of the study protocol must also be provided in the manuscript.

Field-specific reporting

Please select the one below that is the best fit for your research. If you are not sure, read the appropriate sections before making your selection.

[x] Life sciences [ ] Behavioural & social sciences [ ] Ecological, evolutionary & environmental sciences

For a reference copy of the document with all sections, see nature.com/documents/nr-reporting-summary-flat.pdf

Life sciences study design

All studies must disclose on these points even when the disclosure is negative.

Sample size	For calcium imaging, every assay include at least 100 cells.
Data exclusions	Particles grouped into bad classes after 2D and 3D classification were excluded from the final map calculation.
Replication	For calcium imaging, all the assays are repeated at least three times with similar results.
Randomization	Particles were randomly split into 2 groups following gold standard FSC for resolution estimation.
Blinding	Investigators were not blinded during cryo-EM data collection and processing, because visual inspection is necessary for structural determination and analysis in this study.

Reporting for specific materials, systems and methods

We require information from authors about some types of materials, experimental systems and methods used in many studies. Here, indicate whether each material, system or method listed is relevant to your study. If you are not sure if a list item applies to your research, read the appropriate section before selecting a response.

Materials & experimental systems

n/a	Involved in the study	
[ ]	[x]	Antibodies
[ ]	[x]	Eukaryotic cell lines
[x]	[ ]	Palaeontology and archaeology
[x]	[ ]	Animals and other organisms
[x]	[ ]	Clinical data
[x]	[ ]	Dual use research of concern
[x]	[ ]	Plants

Methods

n/a	Involved in the study	
[x]	[ ]	ChIP-seq
[x]	[ ]	Flow cytometry
[x]	[ ]	MRI-based neuroimaging

Antibodies

Antibodies used	GFP tag rabbit polyAb (1:10000 dilution), Proteintech Cat No.#50430-2-AP, Lot#00105240; Monoclonal anti flag M2 antibody produced in mouse (1:10000 dilution), Sigma Cat No. #F1804, Lot#SLBX2256; Goat Anti-Rabbit lgG (1:10000 dilution), TransGen Code#HS101-01, Lot#P21118; Goat Anti-Mouse lgG (1:10000 dilution), TransGen Code#HS201-01, Lot#R10110
Validation	All antibodies in this study were acquired from commercial providers, and validated following manufacturers' guidelines.

Eukaryotic cell lines

Policy information about cell lines and Sex and Gender in Research

Cell line source(s)	HEK293s and sf9 are from Thermo Fisher Scientific. Neuro-2a is from American Type Culture Collection (ATCC).
Authentication	Not performed for commercially available cell lines.
Mycoplasma contamination	Not tested for mycoplasma contamination.
Commonly misidentified lines (See ICLAC register)	No commonly misidentified lines were used.

Plants

Seed stocks	N/A
Novel plant genotypes	N/A
Authentication	N/A
