# Supplementary material for: Cryo-EM structure of TMEM63C suggests it functions as a monomer
Source: Nat Commun. 2023 Nov 9;14:7265. doi: 10.1038/s41467-023-42956-2 (PMC10636204; doi:10.1038/s41467-023-42956-2)
Supplement: Supplementary file 2 — Peer Review Filepdf [file 41467_2023_42956_MOESM2_ESM.pdf]

nature portfolio

# Peer Review File

## Cryo-EM structure of TMEM63C suggests it functions as a monomer

**Open Access** This file is licensed under a Creative Commons Attribution 4.0 International License, which permits use, sharing, adaptation, distribution and reproduction in any medium or format, as long as you give appropriate credit to the original author(s) and the source, provide a link to the Creative Commons license, and indicate if changes were made. In the cases where the authors are anonymous, such as is the case for the reports of anonymous peer reviewers, author attribution should be to 'Anonymous Referee' followed by a clear attribution to the source work. The images or other third party material in this file are included in the article's Creative Commons license, unless indicated otherwise in a credit line to the material. If material is not included in the article's Creative Commons license and your intended use is not permitted by statutory regulation or exceeds the permitted use, you will need to obtain permission directly from the copyright holder. To view a copy of this license, visit http://creativecommons.org/licenses/by/4.0/.

Reviewers' Comments:

Reviewer #1:
Remarks to the Author:
In manuscript#: NCOMMS-23-30634 titled "Cryo-EM structure of TMEM63C suggests it functions as a monomer", Yuqi Qin et al reported the monomer Cryo-EM structure of mTMEM63C, and proposed that oligomerization for TMEM63 proteins to be a potential novel regulatory mechanism, along with elucidating the important roles of coupling between TM0 and TM6 in channel activity. Meanwhile, the author used the biochemical method to demonstrate the physiological state of mTMEM63C is indeed a monomeric state.
This result is interesting, but maybe it can be more exciting to see the Cryo-EM structures of the mix of monomer and dimer of TMEM63B as the author claimed in the paper for further proper elucidation of the mechanisms.

Major Comment #1: The experimental structure determination and data processing methods were sufficient for providing the 3D model. Along with the detailed experimental procedures, the results were well presented along with the necessary sound reasoning behind stated results. However, there is a missing article by Mingfeng Zhang and his colleague's work, titled: 'A mechanical-coupling mechanism in OSCA/TMEM63 channel mechanosensitive'. This missing article contains the monomer hTMEM63A structure. Therefore, it's necessary to cite the aforementioned article and the authors need to add the results of comparing structure, mechanism, or function with mTMEM63C. Overall, the structural results were cohesive in Nature Communications but might need an urgent update concerning the recent development in the field.

Major Comment #2: The authors utilized the cytoplasmic Ca2+ influx responding ratio of OSCA/TMEM63 expressing cells to hypo-osmolality stress to indicate the channel activity. Yes, despite it is difficult to record the negative pressure-activated currents due to the high threshold, the alternative way of channel activity indicator, for me, should remain cautious. At least, the author should provide evidence to show that there is a direct relationship between the cytoplasmic Ca2+ influx responding ratio and channel activity.

Minor Comments:
The manuscript is relatively well written, but it would have been better if providing more background information about mechanosensitive ion channels, especially about the structural achievements.
The manuscript misinterprets the contribution of OSCA channels as mechanosensitive ion channels. As the "gold" standard method for identifying mechanosensitive ion channels is negative pressure activation (e.g., bacterial MscL and MscS channels) or poking activation (e.g., Piezo channels), Zhang et al. first identified and reported OSCA is the high threshold mechanical force-activated channel comparison to the Piezo channel in the NSMB paper (Zhang, M. et al. Structure of the mechanosensitive OSCA channels. Nat. Struct. Mol. Biol. 25, 850–858 (2018).). The author should carefully cite the paper throughout the manuscript.

There are no particular ethical concerns for the structural work, despite more structural results may further enhance the work. This manuscript merits consideration for publication in Nature Communications as an original research Article. We recommend that this manuscript could be published after addressing the major and minor concerns.

Reviewer #2:
Remarks to the Author:
In this manuscript, Qin et al. present the cryo-EM structure of TMEM63C in a closed conformation at 3.5 Å resolution. The structural analysis reveals key residues that are important for channel activity, which is further corroborated by calcium imaging experiments. The authors also investigate the role of

TM0 and TM6 coupling in the regulation of channel activity through mutagenesis and biochemical assays. Interestingly, unlike its plant orthologs OSCAs, TMEM63C exists as a monomer, as confirmed by both cryo-EM structure and biochemical assays, while TMEM63B exhibits a mixture of monomeric and dimeric forms. Based on these findings, the authors propose that oligomerization may regulate the channel activity of TMEM63 proteins, providing valuable insights for future studies on the working mechanism of TMEM63 proteins.

In summary, this manuscript presents solid structural studies and investigates the function of TMEM63B through homology modeling, given its high sequence similarity with TMEM63C. The main conclusions are further supported by mutagenesis and biochemical assays. The findings in this manuscript offer valuable insights for future studies on the mechanisms of TMEM16 superfamily proteins.

Following please find my comments:

Major:

1. The authors used a hypotonic solution with a salt concentration of 170 mM NaCl to measure calcium signals. Will such a low salt concentration cause cell to lysate or burst as reported before (PMID: 23127534)? It should be noted that calcium will enter the cells when cells rupture and will induce very high (saturated) calcium response (false positive).
2. In the manuscript, the authors used the number of responding cells as an indicator of TMEM63C protein activity. Measurement of EC50 by electrophysiological recording or DeltaF/F may provide more accurate results.
3. Hypotonic stimulation can induce a variety of signaling such as arachidonic acid metabolites and ROS (PMID: 19126758), these signaling might be involved in the hypertonic induced calcium response in current study, this is consistent with the large and inconsistent delay in calcium response in the authors' previous publication (PMID: 32375046) and current study, the authors should discuss these possibilities.
4. In contrast to monomeric TMEM63C, the authors found that TMEM63B can form both monomer and dimer in the cryo-EM study, which is different from recent publications of the structure of TMEM63B. This observation is interesting and important for understanding the regulatory mechanism of TMEM63 proteins. Are there additional pieces of evidence, besides cryo-EM, to support the observation of dimeric TMEM63B?
5. In the manuscript, the authors used electrophysiological studies to describe the results of "cytoplasmic Ca2+ measurements of transfected cells," which is inappropriate and needs to be corrected.
6. The authors found that the double mutant (V39C and N545C) of TMEM63C exhibited higher activity than the single mutants (V39C, N545C) or wild type, suggesting that the coupling of TM0 and TM6 may play an important role in channel activity. More experiments are needed to support this hypothesis, such as measuring the channel activity of V39C, N545C in the presence of a reducing agent like DTT.
7. TMEM63C mutations of V39 on TM0, Q451 on M4, N545 on TM6, and their conserved corresponding site on TMEM63B (V44、Q477、N571) are critical for channel opening. How about the channel activity of the corresponding mutation in TMEM63B?
8. Recent studies have reported the presence of lipid-like densities in the cryo-EM structures of TMEM63, which may be related to channel activity. Did the authors also observe any lipids in their structure?
9. The authors should include more structure comparisons and analysis of the TMEM63C structure reported in this study with recently published structures of TMEM63A and TMEM63 if their PDB/EMDB entries are available.

Minor：

1. In the legend of Figure 4, the full name of "GA" should be provided.
2. What species is TMEM63B purified in this article? Please make it clear.
3. In addition, the expression and purification method of TMEM63B should be added.
4. In the figure legend of Figure 2, Figure3 and Figure 4, (a)(b)... should be the same font.

**Response to the reviewers' comments:**

We would like to express our appreciation to the reviewers for their constructive and insightful comments. Taking into account their valuable feedback, we have conducted additional experiments and made extensive revisions to the manuscript, addressing all the raised points. As a result, the quality and accessibility of the manuscript have been significantly improved.

Reviewer #1 (Remarks to the Author):

In manuscript#: NCOMMS-23-30634 titled "Cryo-EM structure of TMEM63C suggests it functions as a monomer", Yuqi Qin et al reported the monomer Cryo-EM structure of mTMEM63C, and proposed that oligomerization for TMEM63 proteins to be a potential novel regulatory mechanism, along with elucidating the important roles of coupling between TM0 and TM6 in channel activity. Meanwhile, the author used the biochemical method to demonstrate the physiological state of mTMEM63C is indeed a monomeric state.

This result is interesting, but maybe it can be more exciting to see the Cryo-EM structures of the mix of monomer and dimer of TMEM63B as the author claimed in the paper for further proper elucidation of the mechanisms.

Thanks the reviewer for the positive comments regarding our manuscript. As highlighted in the 2D averages of our cryo-EM study (*Supplementary Figure 7b*), along with additional biochemical assays (*Figure 4d*) included in the revised manuscript, we have indeed observed the formation of dimers by TMEM63B, albeit at a low percentage. We appreciate the reviewer's suggestion of capturing TMEM63B in both monomer and dimer conformations, which would be an exciting prospect. To address this suggestion, we are currently optimizing experimental conditions in order to increase the percentage of dimeric TMEM63B. This will enable us to conduct a high-resolution cryo-EM study with a greater representation of the dimeric form. The subsequent investigations will shed further light on the regulatory mechanisms governing TMEM63B.

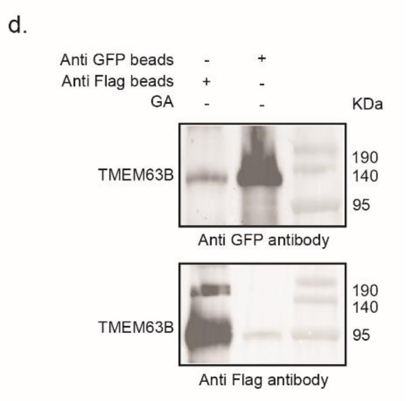


*Figure 4 Native state of TMEM63C is a monomer. (**d**) Co-IP of TMEM63B without crosslinking.*

Major Comment #1: The experimental structure determination and data processing methods were sufficient for providing the 3D model. Along with the detailed experimental procedures, the results were well presented along with the necessary sound reasoning behind stated results. However, there is a missing article by Mingfeng Zhang and his colleague's work, titled: 'A mechanical-coupling mechanism in

OSCA/TMEM63 channel mechanosensitive'. This missing article contains the monomer hTMEM63A structure. Therefore, it's necessary to cite the aforementioned article and the authors need to add the results of comparing structure, mechanism, or function with mTMEM63C. Overall, the structural results were cohesive in Nature Communications but might need an urgent update concerning the recent development in the field.

Thanks for the positive feedback and pointing this out. During the review process of our manuscript, two studies of TMEM63 proteins have been published. In response, we conducted a comprehensive comparison between our structure and the structures reported by these two recent studies[1,2]. In the revised manuscript, we present the results of this structural comparison. Our analysis revealed that the overall structure of mTMEM63C closely resembles that of hTMEM63s, with slight differences observed in TM6b and TM10 (*Supplementary Figure 6*). Additionally, we identified two non-protein densities within our cryo-EM map of TMEM63C, which are likely composed of lipids. These lipid densities are surrounded by specific residues in TM5 and TM9, situated in close proximity to the lipid observations made in the structures of hTMEM63A and TMEM63B[2] (*Supplementary Figure 3c*). To address these observed differences and explore their potential implications for the protein's function, we have included an additional paragraph in the revised manuscript (Lines 234-251).

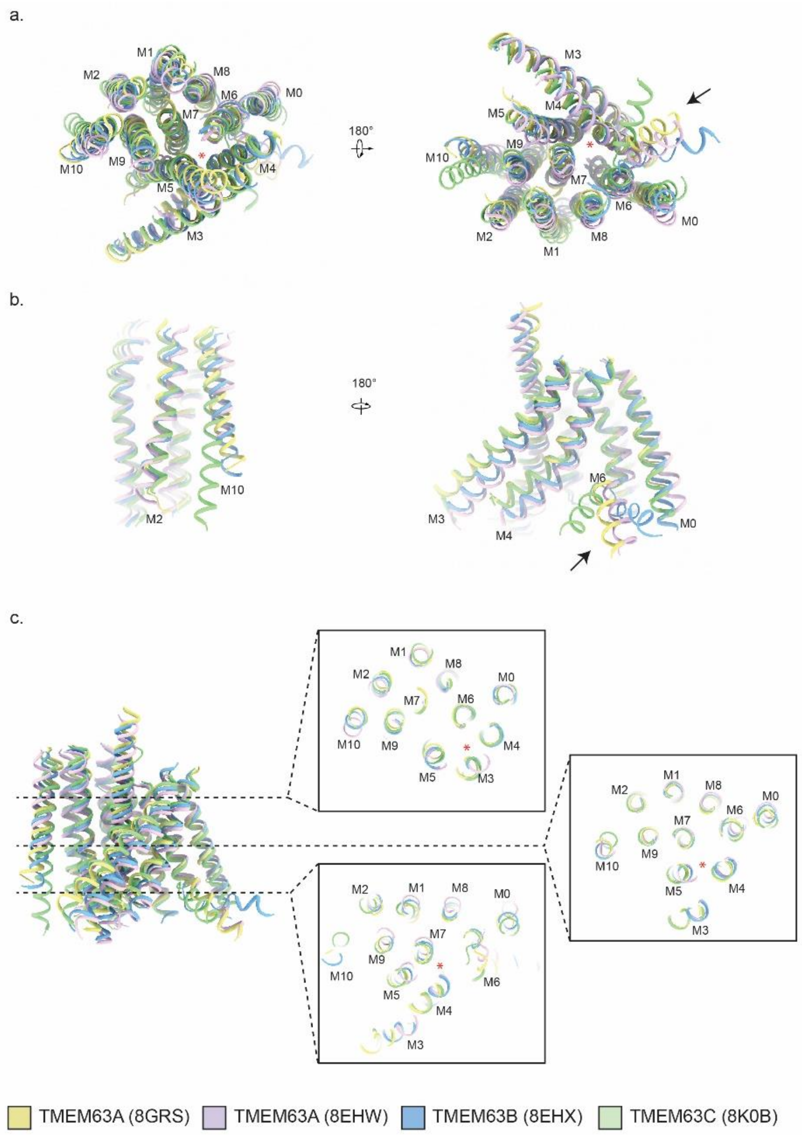


Supplementary Figure 6 *Structural comparison of TMEM63s. (**a**) Top view (left) and bottom view (right) of TMEM63A (yellow, PDB: 8GRS), TMEM63A (pink, PDB: 8EHW), TMEM63B (blue, PDB: 8EHX), and TMEM63C (green, PDB: 8K0B). The eleven transmembrane α-helices are labeled with M0 to M10. (**b**) Side views of TMEM63. The M10, M3, M4 and M6b (black arrows) show conformation changes. (**c**) Cross sections of TMEM63. The red asterisk indicates the pore region.*

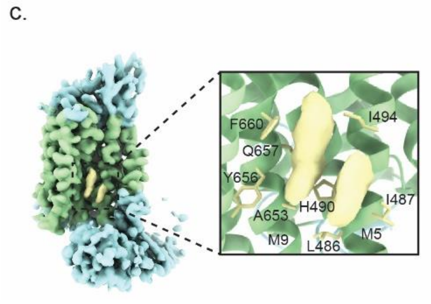


Supplementary Figure 3 *Cryo-EM analysis of TMEM63C. (**c**) Side view of TMEM63C. The lipid-like density is colored in yellow. The lipid surrounding residues in TM5 and*

*TM9 are shown as yellow.*

Major Comment #2: The authors utilized the cytoplasmic $Ca^{2+}$ influx responding ratio of OSCA/TMEM63 expressing cells to hypo-osmolality stress to indicate the channel activity. Yes, despite it is difficult to record the negative pressure-activated currents due to the high threshold, the alternative way of channel activity indicator, for me, should remain cautious. At least, the author should provide evidence to show that there is a direct relationship between the cytoplasmic $Ca^{2+}$ influx responding ratio and channel activity.

Thank the reviewer for the thoughtful consideration. As mentioned by the reviewer, directly measuring the channel currents electrophysiologically for TMEM63 channels is challenging due to their low channel activity and high mechanical threshold[2,3]. In our previous study, we found that activation of TMEM63 channels by hypotonic stress triggers extracellular $Ca^{2+}$ influx[4]. The percentage of cells responding to hypotonic stress serves as a sensitive measure (*Figure R1*, (Figure S1G from Du, H. *et al*, 2020)[4]), which positively correlates with the channel currents induced by negative pressure from Adrem Patapoutian group (*Figure R2*, (Figure 4C from Murthy, S.E. *et al*, 2020)[3]). These results suggest that osmosensitivity reflects mechanosensitivity.

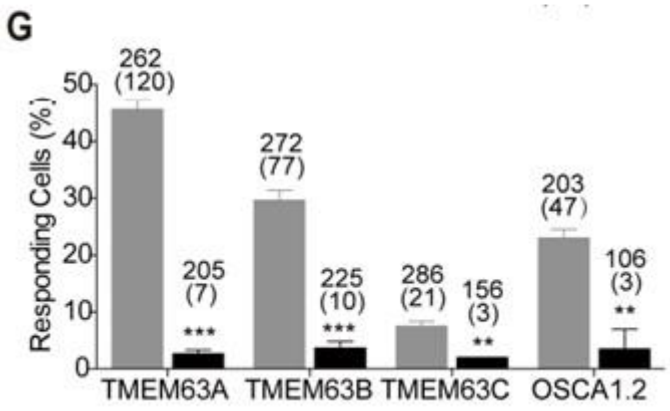


*Figure R1 TMEM63/OSCA Family Mediate Calcium Influx in Response to Hypertonic and Hypotonic Stimuli. (**G**) The percentage of cells transfected with different TMEM63/OSCA homolo -gs responding to hypo- or hyperosmotic stimuli. (**excerpted from Du, H. et al, 2020**)[4].*

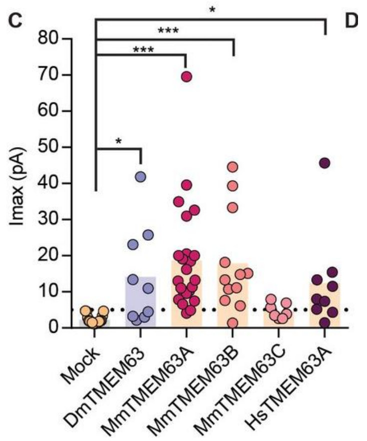


*Figure R2 OSCA orthologues in flies and in mammals induce MA currents in HEK-P1KO cells. (**C**) Maximal stretch-activated currents recorded from cells expressing*

*mock plasmid (N = 17) or the indicated TMEM63 homologue (DmTMEM63 (N = 7), MmTMEM63A (N = 23), MmTMEM63B (N = 12), MmTMEM63C (N = 7), and HsTMEM63A (N = 6). DmTMEM63: *p=0.023, HsTMEM63A: *p=0.015, ***p<0.0001, Dunn's multiple comparison test relative to mock plasmid). (**excerpted from Murthy, S.E. et al, 2018**)[3].*

To study the mechanism of TMEM63 proteins, we opted for measuring cytoplasmic $Ca^{2+}$ influx as an alternative approach. This method offers simplicity and reliability in reflecting channel activity. However, we fully agree with the reviewer that the responding ratio is not a direct measurement of the channel activity. Therefore, in the revised manuscript (Lines 148, 160), we have included measurements of the maximum $Ca^{2+}$ rise ($\Delta F/F0$), which provides a more direct reflection of channel activity (*Figure 2f,i, Figure 3c,f*). The results from these measurements also demonstrate a generally positive correlation between the responding ratio and channel activity.

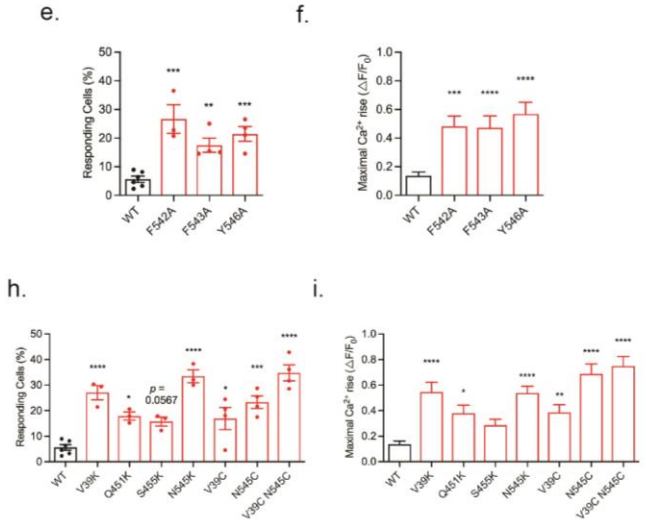


*Figure 2 The pore profile and TM0-TM6 coupling of TMEM63C. (**e**) The responding ratio TMEM63C expressing cells to hypo-osmolality stress. (**f**) The maximal $Ca^{2+}$ rise in response to hypo-osmolality stress. (**h**) The responding ratio TMEM63C expressing cells to hypo-osmolality stress. (**i**) The maximal $Ca^{2+}$ rise in response to hypo-osmolality stress. Data (e, f, h, i) are shown as mean ± SEM. *p < 0.05; **p < 0.01; ***p < 0.001; ****p < 0.0001; one-way ANOVA followed by Tukey's multiple comparisons test.*

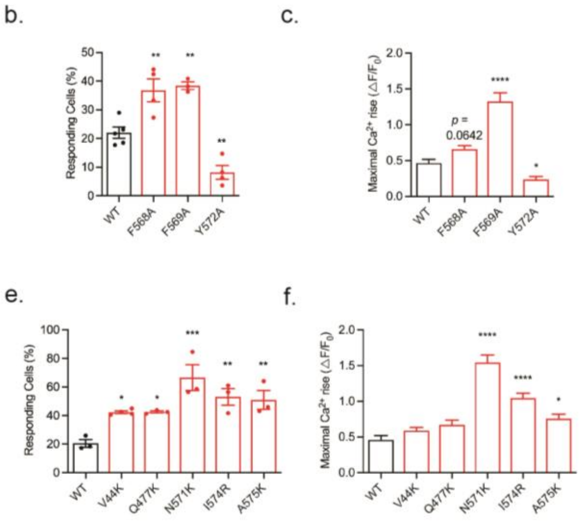


*Figure 3 TMEM63B channel activity effected by the conserved residues identified in TMEM63C. (**b**) The responding ratio of TMEM63B variants expressing cells to hypo-osmolality stress. (**c**) The maximal $Ca^{2+}$ rise of TMEM63B variants in response to hypo-osmolality stress. (**e**) The responding ratio of TMEM63B variants expressing cells to hypo-osmolality stress. (**f**) The maximal Ca2+ rise of TMEM63B variants in response to hypo-osmolality stress. Data (b, c, e, f) are shown as mean ± SEM. *p < 0.05; **p < 0.01; ***p < 0.001; ****p < 0.0001; one-way ANOVA followed by Tukey's multiple comparisons test.*

Minor Comments:

The manuscript is relatively well written, but it would have been better if providing more background information about mechanosensitive ion channels, especially about the structural achievements.

The manuscript misinterprets the contribution of OSCA channels as mechanosensitive ion channels. As the "gold" standard method for identifying mechanosensitive ion channels is negative pressure activation (e.g., bacterial MscL and MscS channels) or poking activation (e.g., Piezo channels), Zhang et al. first identified and reported OSCA is the high threshold mechanical force-activated channel comparison to the Piezo channel in the NSMB paper (Zhang, M. et al. Structure of the mechanosensitive OSCA channels. Nat. Struct. Mol. Biol. 25, 850–858 (2018).). The author should carefully cite the paper throughout the manuscript.

We apologize for the misinterpretation. In the revised manuscript, we provided a brief introduction of mechanosensitive ion channels and cited related literatures appropriately.

There are no particular ethical concerns for the structural work, despite more structural results may further enhance the work. This manuscript merits consideration for publication in Nature Communications as an original research Article. We recommend that this manuscript could be published after addressing the major and minor concerns.

We thank the reviewer for providing positive feedback and valuable constructive suggestions to enhance our manuscript.

Reviewer #2 (Remarks to the Author):

In this manuscript, Qin et al. present the cryo-EM structure of TMEM63C in a closed conformation at 3.5 Å resolution. The structural analysis reveals key residues that are important for channel activity, which is further corroborated by calcium imaging experiments. The authors also investigate the role of TM0 and TM6 coupling in the regulation of channel activity through mutagenesis and biochemical assays. Interestingly, unlike its plant orthologs OSCAs, TMEM63C exists as a monomer, as confirmed by both cryo-EM structure and biochemical assays, while TMEM63B exhibits a mixture of monomeric and dimeric forms. Based on these findings, the authors propose that oligomerization may regulate the channel activity of TMEM63 proteins, providing valuable insights for future studies on the working mechanism of TMEM63 proteins.

In summary, this manuscript presents solid structural studies and investigates the function of TMEM63B through homology modeling, given its high sequence similarity with TMEM63C. The main conclusions are further supported by mutagenesis and biochemical assays. The findings in this manuscript offer valuable insights for future studies on the mechanisms of TMEM16 superfamily proteins.

We thank the reviewer for the positive comments and constructive suggestions of our manuscript.

Following please find my comments:

Major:

1. The authors used a hypotonic solution with a salt concentration of 170 mM NaCl to measure calcium signals. Will such a low salt concentration cause cell to lysate or burst as reported before (PMID: 23127534)? It should be noted that calcium will enter the cells when cells rupture and will induce very high (saturated) calcium response (false positive).

Thanks the reviewer for this thoughtful concern. Continuous exposure to hypotonic stress may potentially cause bursts in certain cell types, including the pancreatic cancer cells as mentioned by the reviewer[5]. In our previous study, we demonstrated that N2a cells can tolerate a hypotonic challenge of 170 mOsm/L without experiencing cell rupture. Specifically, under 170 mOsm/L hypotonic stress conditions, we observed a significant calcium response exclusively in TMEM63B-expressing cells, while the control cells expressing GCaMP6f did not exhibit such a response (*Figure R3* (Figure 1A, E-F from Du, H. *et al*, 2020)[4]). Therefore, in this study, we used N2a cells to investigate the mechanism of TMEM63 by measuring their calcium response. We hope this explanation addresses the reviewer's concern.

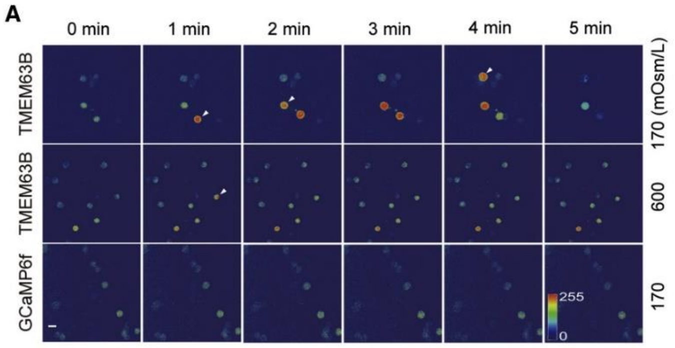

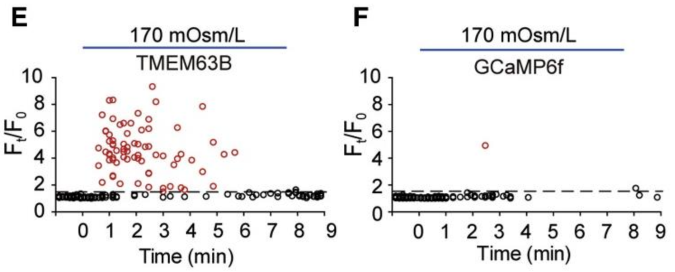


*Figure R3 TMEM63B mediates $Ca^{2+}$ influx in response to hypotonic stimulus. (**A**) Fluorescent emission images of N2a cells transfected with TMEM63B-P2A GCaMP6f, in response to 600 mOsm/L and 170 mOsm/L solutions. White arrowheads marked the responding cells. Scale bar: 10 μm. (**E**) The maximal $Ca^{2+}$ fluorescence intensity in response to 170 mOsm/L solutions was plotted to exposure time. The data were pooled from three experiments. (**F**) The maximal responses of cells transfected with GCaMP6f challenged by 170 mOsm/L solution were plotted to exposure time. (**excerpted from Du, H. et al, 2020**)[4].*

2. In the manuscript, the authors used the number of responding cells as an indicator of TMEM63C protein activity. Measurement of EC50 by electrophysiological recording or DeltaF/F may provide more accurate results.

Thanks for pointing this out. The reviewer 1 also raised a similar concern (Major Concern 2). In our previous study, we have successfully demonstrated a positive correlation between channel activity and number of responding cells for TMEM63 proteins. Furthermore, in response to this concern, we have included ΔF/F0 as a more direct measurement of the channel activity in the revised manuscript (*Figure 2f, i, Figure 3c,f*), which provides clearer insights into the functional characteristics of the channels.

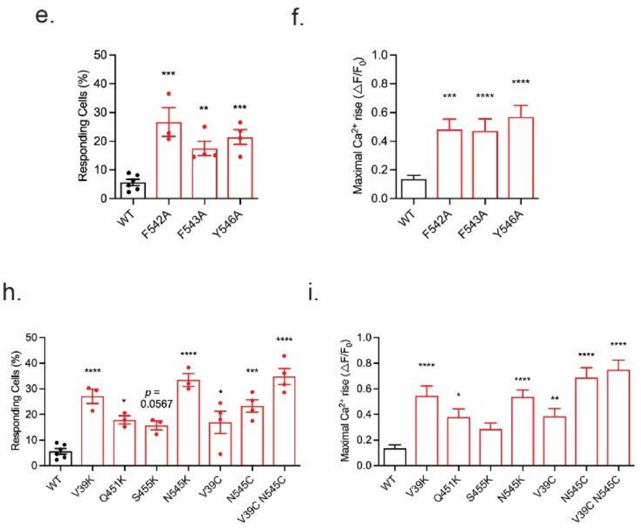


*Figure 2 The pore profile and TM0-TM6 coupling of TMEM63C. (**e**) The responding ratio TMEM63C expressing cells to hypo-osmolality stress. (**f**) The maximal $Ca^{2+}$ rise in response to hypo-osmolality stress. (**h**) The responding ratio TMEM63C expressing cells to hypo-osmolality stress. (**i**) The maximal $Ca^{2+}$ rise in response to hypo-osmolality stress. Data (e, f, h, i) are shown as mean ± SEM. *p < 0.05; **p < 0.01;*

****p < 0.001; ****p < 0.0001; one-way ANOVA followed by Tukey's multiple comparisons test.*

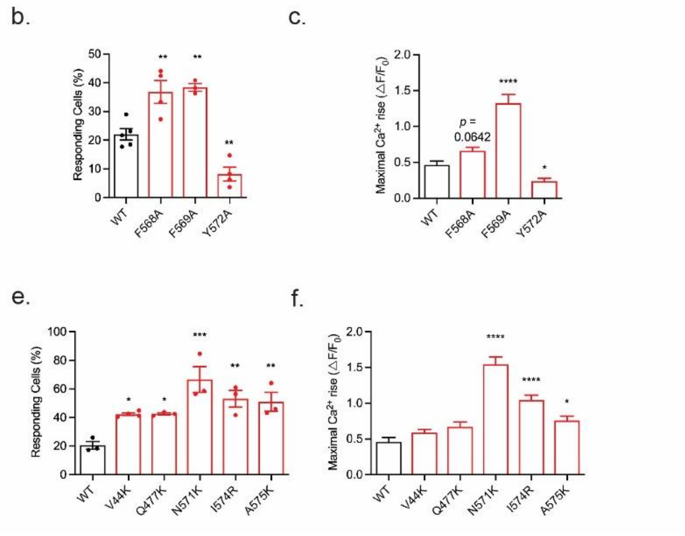


*Figure 3 TMEM63B channel activity effected by the conserved residues identified in TMEM63C. (**b**) The responding ratio of TMEM63B variants expressing cells to hypo-osmolality stress. (**c**) The maximal $Ca^{2+}$ rise of TMEM63B variants in response to hypo-osmolality stress. (**e**) The responding ratio of TMEM63B variants expressing cells to hypo-osmolality stress. (**f**) The maximal Ca2+ rise of TMEM63B variants in response to hypo-osmolality stress. Data (b, c, e, f) are shown as mean ± SEM. *p < 0.05; **p < 0.01; ***p < 0.001; ****p < 0.0001; one-way ANOVA followed by Tukey's multiple comparisons test.*

3. Hypotonic stimulation can induce a variety of signaling such as arachidonic acid metabolites and ROS (PMID: 19126758), these signaling might be involved in the hypertonic induced calcium response in current study, this is consistent with the large and inconsistent delay in calcium response in the authors' previous publication (PMID: 32375046) and current study, the authors should discuss these possibilities.

Thanks the reviewer for this critical comment and inspiring question. It seems that the delayed response of ion channels to hypotonic or hypertonic stress is a common phenomenon. In addition to our studies on TMEM63s, other research groups also reported similar observations on ion channels including TRPC5[6] (*Figure R4* (Figure 2A from Gomis, A. et al, 2008)). We fully agree with the reviewer that hypotonic stress may contribute to the regulation of the channel activity, leading to a delayed and inconsistent calcium response. We have discussed this possibility in the revised manuscript (Lines 263-273). More works are needed to unravel the underlying mechanisms behind the observed delayed response and further enhance our understanding of TMEM63 function during hypotonic stress conditions.

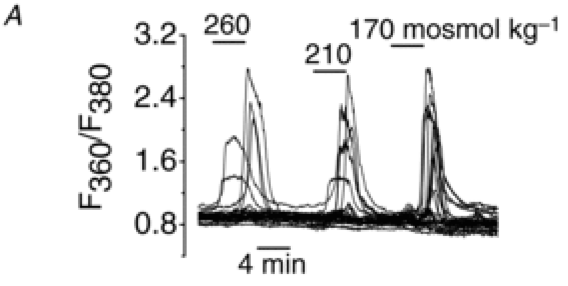

*Figure R4 Osmolarity dependence of responsive cells in fura-2-loaded HEK293 cells expressing TRPC5. (**A**) [$Ca^{2+}$]i changes in response to reduction in extracellular osmolarity from 300 mosmol $kg^{-1}$ to the indicated values (for these set of experiments the fura-2 fluorescence was measured at its isosbestic point). (**excerpted from Gomis, A. et al, 2008**)[6].*

4. In contrast to monomeric TMEM63C, the authors found that TMEM63B can form both monomer and dimer in the cryo-EM study, which is different from recent publications of the structure of TMEM63B. This observation is interesting and important for understanding the regulatory mechanism of TMEM63 proteins. Are there additional pieces of evidence, besides cryo-EM, to support the observation of dimeric TMEM63B?

Thanks for the reviewer's suggestion. Following the same protocol, we performed a pull-down assay to examine the oligomerization state for TMEM63B in cells. Specifically, the cells co-expressing of TMEM63B-GFP and TMEM63B-FLAG were solubilized with DDM, and loaded onto GFP or FLAG beads, respectively. The results of western blotting analysis revealed that samples from the FLAG beads could be detected by both the GFP and FLAG antibodies (*Figure 4d*, left lanes), although the signals detected by GFP antibody were much weaker. Similar results were observed for samples from the GFP beads (*Figure 4d*, middle lanes). These results suggested the existence of oligomeric TMEM63B. Combined with our cryo-EM observations, these findings further support that TMEM63B exists as a mixture of monomers and dimers. We have added the pull-down assay of TMEM63B in the revised manuscript. (Lines 291-296)

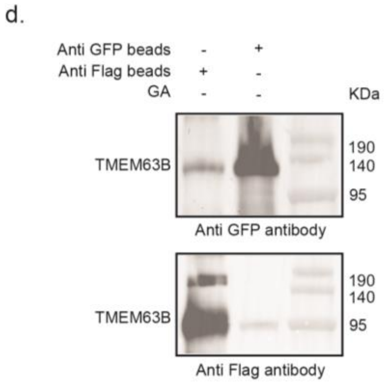


*Figure 4 Native state of TMEM63C is a monomer. (**d**) Co-IP of TMEM63B without crosslinking.*

5. In the manuscript, the authors used electrophysiological studies to describe the results of "cytoplasmic $Ca^{2+}$ measurements of transfected cells," which is inappropriate and needs to be corrected.

Points taken. We have addressed this misinterpretation by making the necessary corrections in the revised manuscript. Specifically, we have replaced the "electrophysiological studies" with "calcium imaging" or "functional analysis" to accurately reflect the experimental approach used in our study (Lines 33, 86, 149, 194, and 675).

6. The authors found that the double mutant (V39C and N545C) of TMEM63C exhibited higher activity than the single mutants (V39C, N545C) or wild type, suggesting that the coupling of TM0 and TM6 may play an important role in channel

activity. More experiments are needed to support this hypothesis, such as measuring the channel activity of V39C, N545C in the presence of a reducing agent like DTT.

Thanks for the reviewer's suggestion. We conducted calcium imaging experiments on the mutant variants, both in the presence and absence of the reducing agent (DTT). Notably, when DTT was added, the channel activity of the double mutant (V39C, N545C) TMEM63C significantly decreased. In contrast, the channel activity of the wild type or single mutant (N545C) TMEM63C remained unchanged upon DTT treatment (*Figure 2j-k*).

These results further strengthen our conclusion that the coupling between TM0 and TM6 plays a crucial role in the channel activity of TMEM63 proteins. We have incorporated these results into the revised manuscript (Lines 171-176).

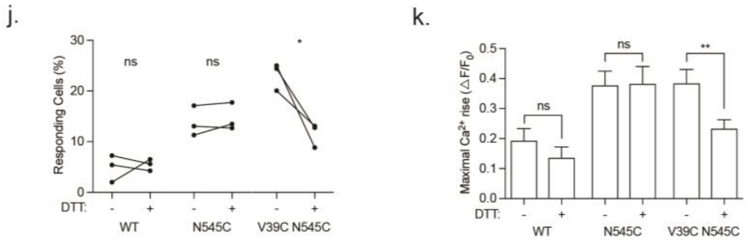


*Figure 2 The pore profile and TM0-TM6 coupling of TMEM63C. (**j-k**) The channel activity of double mutant (V39C N545C) but not wild type or single mutant (N545C) TMEM63C decreased significantly in the presence of DTT. The responding cells (j) and maximal $Ca^{2+}$ rise (k) in response to hypo-osmolality stress are shown. Data (j, k) are shown as mean ± SEM. *p < 0.05; **p < 0.01; ns, not significant; paired (j) or unpaired (k) two-tailed t test.*

7. TMEM63C mutations of V39 on TM0, Q451 on M4, N545 on TM6, and their conserved corresponding site on TMEM63B (V44、Q477、N571) are critical for channel opening. How about the channel activity of the corresponding mutation in TMEM63B?

Thanks for the reviewer's suggestion. In order to further confirm the critical roles of these conserved residues identified in TMEM63C, we conducted measurements of channel activity using corresponding mutants (V44K, Q477K, and N571K) in TMEM63B through calcium imaging. In line with our observation in TMEM63C, these three mutants of TMEM63B demonstrated a higher responding ratio compared to the wild type (*Figure 3d-f*). We have included these results in the revised manuscript (Lines 192-201).

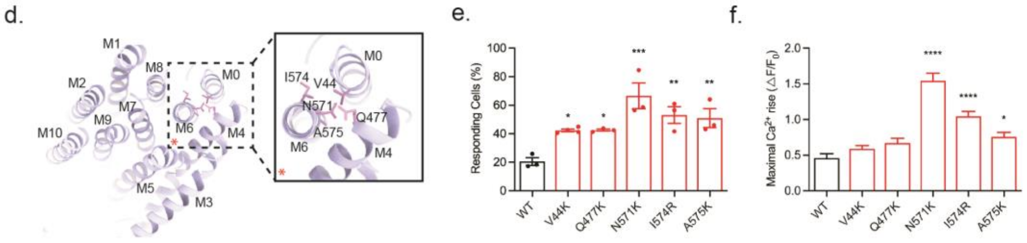


*Figure 3 TMEM63B channel activity effected by the conserved residues identified in TMEM63C. (**d**) The TMEM63B residues V44 in M0, Q477K in M4, N571, I574 and A575 in M6 (pink) outside the pore. The position of the pore is indicated with a red asterisk. (**e**) The responding ratio of TMEM63B variants expressing cells to hypo-*

*osmolality stress. (**f**) The maximal $Ca^{2+}$ rise of TMEM63B variants in response to hypo-osmolality stress. Data (e, f) are shown as mean ± SEM. *p < 0.05; **p < 0.01; ***p < 0.001; ****p < 0.0001; one-way ANOVA followed by Tukey's multiple comparisons test.*

8. Recent studies have reported the presence of lipid-like densities in the cryo-EM structures of TMEM63, which may be related to channel activity. Did the authors also observe any lipids in their structure?

Thanks for pointing this out. We indeed observed two non-protein densities, which are likely composed of lipids, in our cryo-EM map of TMEM63C. These lipids are surrounded by several residues in TM5 and TM9, in close proximity to where lipids were observed in the structures of hTMEM63A and TMEM63B[2] (*Supplementary Figure 3c*). We have added this information in the revised manuscript (Line 246-251).

c.

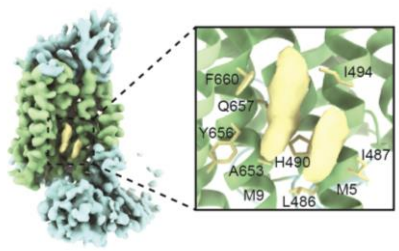


*Supplementary Figure 3 Cryo-EM analysis of TMEM63C. (**c**) Side view of TMEM63C. The lipid-like density is colored in yellow. The lipid surrounding residues in TM5 and TM9 are shown as yellow.*

9. The authors should include more structure comparisons and analysis of the TMEM63C structure reported in this study with recently published structures of TMEM63A and TMEM63 if their PDB/EMDB entries are available.

Thanks the reviewer for this constructive suggestion. While our manuscript was under review, structures of human TMEM63A and TMEM63B have been reported by two groups. In response to the reviewer's suggestion, we conducted a comprehensive structural analysis comparing mTMEM63C (in this study) with hTMEM63A and hTMEM63B[1,2]. Our structural comparison revealed that the overall structure of mTMEM63C is similar to that of hTMEM63s, with slight differences observed in TM6b and TM10 (*Supplementary Figure 6*). To address these differences and their potential relationship with the protein's function, we have included an additional paragraph in the revised manuscript (Lines 234-251).

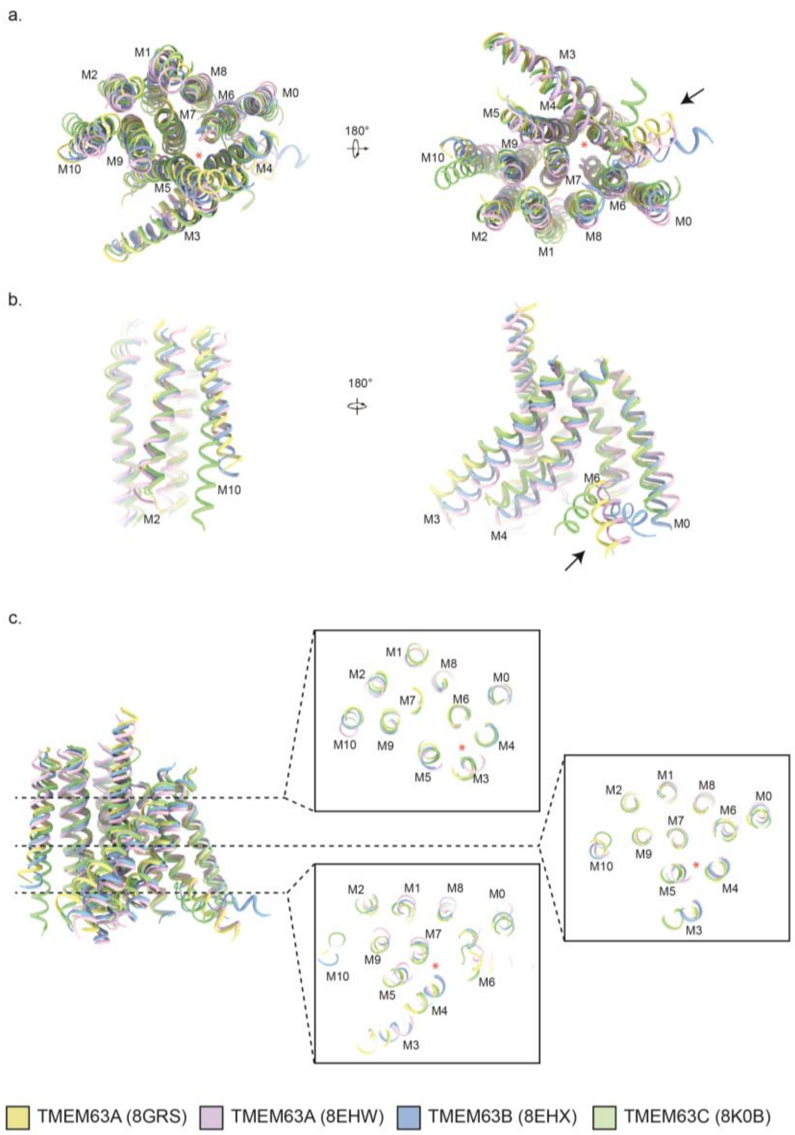


*<u>Supplementary Figure 6</u> Structural comparison of TMEM63s. (a) Top view (left) and bottom view (right) of structural comparison includingTMEM63A (yellow, PDB: 8GRS), TMEM63A (pink, PDB: 8EHW), TMEM63B (blue, PDB: 8EHX), and TMEM63C (green, PDB: 8K0B). The eleven transmembrane α-helices are labeled with M0 to M10. (b) Side views of TMEM63. The M10, M3, M4 and M6b (black arrows) show conformation changes. (c) Cross sections of structural comparison of TMEM63 proteins. The red asterisk indicates the pore region.*

Minor:

1. In the legend of Figure 4, the full name of "GA" should be provided.

Point taken. We have made the necessary correction and included the full name of GA (glutaraldehyde) in the revised manuscript (Lines 216, 357).

2. What species is TMEM63B purified in this article? Please make it clear.

We apologize for the confusion. To clarify, we use mouse TMEM63B in this study. We have made this explicitly clear in the revised manuscript (Lines 181, 287, and 542).

3. In addition, the expression and purification method of TMEM63B should be added.

Point taken. The expression and purification of TMEM63B have been included in the method section (Lines 542-553).

4. In the figure legend of Figure 2, Figure3 and Figure 4, (a)(b)... should be the same font.

Points taken. These have been corrected in the revised manuscript (Lines 308-360).

Reference

1. Zhang, M., Shan, Y., Cox, C. D. & Pei, D. A mechanical-coupling mechanism in OSCA/TMEM63 channel mechanosensitivity. *Nat. Commun.* **14**, 3943 (2023).

2. Zheng, W. *et al.* TMEM63 proteins function as monomeric high-threshold mechanosensitive ion channels. *Neuron* (2023). doi:10.1016/J.NEURON.2023.07.006

3. Murthy, S. E. *et al.* OSCA/TMEM63 are an evolutionarily conserved family of mechanically activated ion channels. *Elife* **7**, (2018).

4. Du, H. *et al.* The Cation Channel TMEM63B Is an Osmosensor Required for Hearing. *Cell Rep.* **31**, 107596 (2020).

5. Nako, Y. *et al.* Enhancement of the cytocidal effects of hypotonic solution using a chloride channel blocker in pancreatic cancer cells. *Pancreatology* **12**, 440–448 (2012).

6. Gomis, A., Soriano, S., Belmonte, C. & Viana, F. Hypoosmotic- and pressure-induced membrane stretch activate TRPC5 channels. *J. Physiol.* **586**, 5633–5649 (2008).

Reviewers' Comments:

Reviewer #1:
Remarks to the Author:
I have no further comments.

Reviewer #2:
Remarks to the Author:
The revision answered most of my concerns.
